# Mesenchymal Stem Cells Improve Glycometabolism and Liver Regeneration in the Treatment of Post-hepatectomy Liver Failure

**DOI:** 10.3389/fphys.2019.00412

**Published:** 2019-04-10

**Authors:** Hao-ran Ding, Jing-lin Wang, Zhen-ting Tang, Yue Wang, Guang Zhou, Yang Liu, Hao-zhen Ren, Xiao-lei Shi

**Affiliations:** ^1^Department of Hepatobiliary Surgery, Nanjing Drum Tower Hospital Clinical College of Nanjing Medical University, Nanjing, China; ^2^Department of Hepatobiliary Surgery, The Affiliated Drum Tower Hospital of Nanjing University Medical School, Nanjing, China

**Keywords:** mesenchymal stem cells, hepatectomy, glycogen synthesis, cell transplantation, liver regeneration

## Abstract

**Background:**

The mortality rate of post-hepatectomy liver failure (PHLF) remains very high, and liver transplantation is the only effective treatment regimen for PHLF. Cell transplantation is a potential treatment for liver diseases. Previous studies have proved that mesenchymal stem cells (MSCs) have immunomodulatory functions. In the present study, we found that MSCs promoted glycogen synthesis and liver regeneration in the treatment of PHLF. MSC transplantation also improved the survival rate of rats after 90% partial hepatectomy (PH). In our current study, we aimed to determine the efficacy and mechanism of MSC transplantation in the treatment of PHLF.

**Methods:**

Mesenchymal stem cells were isolated from Sprague-Dawley rats and cultured using a standardized protocol. The MSCs were transplanted to treat acute liver failure induced by 90% PH. The therapeutic efficacy of MSCs on PHLF was verified through measuring alanine transaminase (ALT), aspartate aminotransferase (AST), international normalized ratio (INR), serum ammonia, liver weight to body weight ratio, blood glucose, and histology. To further study the mechanism of MSC transplantation in treatment for PHLF, we assessed the changes in the AKT/glycogen synthase kinase-3β (GSK-3β)/β-catenin pathway. A-674563 (AKT inhibitor) and SB216763 (GSK-3β inhibitor) were employed to validate our findings. SPSS version 19.0 was used for statistical analysis, and the independent-samples *t*-test was carried out to analyze the collected data.

**Results:**

Mesenchymal stem cell transplantation attenuated the liver injury in acute liver failure induced by 90% PH. MSC transplantation improved the glucose metabolism and survival rate in the PHLF model. The effect of MSC transplantation on hepatocyte proliferation might be related to AKT/GSK-3β/β-catenin pathway.

**Conclusion:**

Mesenchymal stem cell transplantation could be use as a potential treatment for PHLF.

## Introduction

Partial hepatectomy is an important treatment for benign and malignant liver diseases. Although the liver can be completely regenerated after partial excision or injury, at least 1/3 of the liver should be retained in most of the patients, and 40–50% should be retained in patients with parenchymal liver disease (Adams et al., 2013; Cieslak et al., 2014). Postoperative complications, such as acute post-hepatectomy liver failure (PHLF) or small liver syndrome, may occur when the scope of excision is too large or the remaining liver is inadequate to maintain normal liver function. As a serious challenge to patient survival, PHLF is the main cause of death after liver surgery (Schreckenbach et al., 2012; Asenbaum et al., 2018). Although surgery and postoperative care have been greatly improved, the incidence of PHLF is between 0.7 and 9% (van den Broek et al., 2008). Therefore, the treatment for PHLF is problematic and needs to be improved.

At present, the most effective therapy for PHLF is liver transplantation. Although liver transplantation has a beneficial therapeutic effect, the insufficient donor liver and the high cost of this operation limit the number of liver transplantations performed (Donnelly et al., 2016). Although symptomatic support therapy can help to prevent the progression of PHLF, it is unsatisfactory for preventing sequelae and improving survival rate (Qadan et al., 2016). Therefore, it is necessary to develop other effective treatments for PHLF.

Alternative therapies for liver transplantation have been investigated, and stem cell therapy is considered to be one of the most promising treatments (Iansante et al., 2018). Stem cell transplantation, as a new treatment method, is widely studied in heart diseases, kidney diseases, nervous system diseases, and liver diseases (Hayes et al., 2015; Tsilimigras et al., 2017; Otsuka et al., 2018; Zare et al., 2018). MSCs are among the most potential stem cells, as they are easily obtained and have advantages of low immunogenicity, self-renewal and multidirectional differentiation (Zhang et al., 2017). Previous studies have shown that MSCs have immunomodulatory functions (Chinnadurai et al., 2018; Ding et al., 2018). Recent studies have demonstrated that MSCs secret soluble cytokines, which inhibit the function of inflammatory cells and prevent excessive inflammatory damage to the liver in a model of drug-induced liver failure. Besides their immunomodulatory functions, these soluble cytokines secreted by MSCs also promote the regeneration of liver. Soluble cytokines, such as IL-6 and TNF-α, which are secreted by MSCs, stimulate liver regeneration after hepatectomy by regulating the hepatocyte proliferation directly or indirectly (Chae et al., 2018; Naseem et al., 2018).

It is known that the liver is an important organ in glycogen metabolism, which is crucial for the energy supply during liver regeneration (Fernández-Rojo et al., 2012). Valdecantos et al. (2017) have reported that increased hepatic glycometabolism can improve liver regeneration after fatty liver resection. Recent research has found that increased hepatic glycogen promotes liver regeneration in an ischemia-reperfusion model (Li et al., 2004). These studies suggest that liver metabolism is directly related to liver regeneration. Tautenhahn et al. (2016) have found that MSCs affect liver metabolism after hepatectomy, but the underlying mechanism requires further investigation. To date, little is known about the relationship between MSCs and hepatic glucose metabolism. In the present study, we aimed to explore the effects of MSCs on liver regeneration and examine the relationship between glucose metabolism and liver regeneration in a rat model with 90% PH.

## Materials and Methods

### Animals

Male Sprague-Dawley (SD) rats (weighing 200–300 g, 7–8 weeks old) underwent 90% PH and MSC transplantation. MSCs were extracted from male SD rats (3–4 weeks old, weighing 50–90 g). All animals were obtained from the Animal Experimental Base of Nanjing Drum Tower Hospital Affiliated to Nanjing University. This experiment was approved by the Institutional Animal Care and Use Committee of Nanjing University, Nanjing, China.

### Extraction of MSCs

Mesenchymal stem cells were purified from 3 to 4-week-old SD rats with a previously established procedure Lehmann et al. (2012). Briefly, rats were sacrificed by cervical dislocation, and the soft tissues surrounding femurs, humeral and tibial bones were carefully removed. The marrow cavity was flushed with complete culture medium. Isolated cells were collected, followed by centrifugation at 1,200 rpm for 5 min, and then the cell pellet was resuspended in growth medium containing low-glucose Dulbecco’s modified Eagle’s medium (DMEM, Gibco, Grand Island, NY, United States) supplemented with 10% fetal bovine serum (Sciencell, San Diego, CA, United States), 100 mg/mL streptomycin and 100 U/mL penicillin. The cells were cultured in a humidified atmosphere containing 5% CO_2_ at 37°C. Non-adherent cells were removed after 24 h, and the culture medium was refreshed every 3 days. MSCs of passages 3–6 were used for experiments.

### Surgery and Treatment

The rats were randomly divided into five groups as follows: (1) control group (sham operation), in which rats underwent laparotomy without 90% PH; (2) PBS+PH group; (3) MSCs+PH group; (4) PH+MSCs+A-674563 (AKT inhibitor, 100 mg/kg; Selleck Chemicals) group; and (5) PH+MSCs+A-674563+SB216763 (GSK-3β) inhibitor, 20 mg/kg; Selleck Chemicals] group. All SD rats undergoing liver resection were fixed and sterilized, and the incision was made following ether anesthesia. The left and middle liver lobes were then resected, followed by resection of right lower lobe and right upper lobe. MSCs were injected into the portal vein to determine the therapeutic effect of MSCs after hepatectomy. Briefly, 1 mL PBS containing 5 × 10^6^ MSCs was injected into the portal vein over a 5-min period. At 1 h before hepatectomy, A-674563 was intraperitoneally injected into the animals.

These five groups (sham operation; PH+PBS; PH+MSCs; PH+MSCs+A-674563; and PH+MSCs+ A-674563+SB216763) were analyzed at 6, 12, 24, 48, and 72 h. Six to eight rats were analyzed at each time point in each group. To estimate the survival rate, another 20 rats in each group were used for analysis.

### Blood Sample Analysis

Blood was extracted from the abdominal aorta at 6, 12, 24, 48, and 72 h after hepatectomy. Levels of alanine aminotransferase (ALT), AST, and blood ammonia as well as the prothrombin INR were determined with an automated biochemical analyzer (iMagic-M7; Mindray, Shenzhen, China).

### Western Blotting Analysis

Western blotting analysis was carried out according to a previously published protocol (Lehmann et al., 2012; Tschuor et al., 2016). Primary antibodies used in the present study included GSK-3β (Cell Signaling Technology, Inc.), p-GSK-3β^Ser9^ (Cell Signaling Technology, Inc.), p-GSK-3β^Tyr216^ (Santa Cruz Biotechnology, Inc.), PCK1 (Cell Signaling Technology, Inc.), PCK2 (Cell Signaling Technology, Inc.), AKT (Cell Signaling Technology, Inc.), p-AKT (Cell Signaling Technology, Inc.), non-phospho (active) β-catenin (Cell Signaling Technology, Inc.), c-Myc (Cell Signaling Technology, Inc.), cyclinD1 (Cell Signaling Technology, Inc.), PCNA (Cell Signaling Technology, Inc.) and MMP7 (Cell Signaling Technology, Inc.). GAPDH (Cell Signaling Technology, Inc.) was used as a loading control. The immunoreative bands were visualized with enhanced chemiluminescence (ECL) reagent (Thermo Fisher Scientific, Waltham, MA, United States). Densitometric analysis of signal intensity was performed using Image J software (NIH, Bethesda, MD, United States).

### Qualitative Real-Time Polymerase Chain Reaction (qRT-PCR)

The extraction of RNA, reverse transcription of cDNA and qRT-PCR were performed according to previously published protocols (Lehmann et al., 2012; Tschuor et al., 2016). The primer sequences were shown in Table 1.

**Table 1:** The sequences of the primers.

Gene	Forward Primer (5′-3′)	Reverse Primer (5′-3′)
Ppargc1a	TCTGGGTGGATTGAAGTGG	CCGCTAGCAAGTTTGCCT
PCK1	TTCGGAAGCGGATACGGT	GGGGTTAGTTATGCCCAGGA
GSK3β	ACCATCCTTATCCCTCCTCAC	CCACGGTCTCCAGCATTAGTA
G6PC	GGCATCAATCTCCTCTGGGT	GGTGACGGGGAACTGTTTT
PCK2	ACGGGTAGAAAGCAAGACG	GCATGCATCCTGGGAATC
c-Myc	GAAGAACAAGATGATGAGGAA	GCTGGTGAGTAGAGACAT
cyclinD1	CAGAAGTGCGAAGAGGAGGT	GGCGGATAGAGTTGTCAGTGT
β-catenin	CCCATCTATGAGGGTTACGC	TTTAATGTCACGCACGATTTC
Axin2	CCGCCACCAAGACCTACATA	GCATTTTCCTCCATCACCG

### Histological Staining and Immunohistochemistry

Liver samples were immobilized with 4% paraformaldehyde and then dehydrated. The paraffin-embedded samples were then sectioned into slices (<3 mm thick, three from each liver). The sections were stained with hematoxylin and eosin for pathological examination. Proliferation was assessed by cell nuclear antigen (Ki67) (Aviva Systems Biology, Beijing, China). PAS (Sigma-Aldrich, 395) staining was used to detect the content of glycogen in the liver. P-AKT, GSK-3β, β-catenin, PCNA and cyclinD1 staining (Previously mentioned) were also carried out. The results represented at least three images per liver. Image analysis and semi-quantification were conducted using image Pro Plus software (Media Cybernetics, Bethesda, MD, United States). Ki67- and PCNA-positive hepatocytes were blindly quantified by manual counting in 10 randomly selected visual fields. Semi-quantification of p-AKT and GSK3β was carried out using image Pro Plus software (Media Cybernetics, Bethesda, MD, United States).

### Statistical Analysis

All data were presented as mean ± standard deviation. The differences between different groups were analyzed by the Student’s *t*-test. The survival rate in rats undergoing 90% PH was analyzed with 20 animals per group. A *p* < 0.05 was considered as statistically significant. Prism software was employed for the statistical analysis (version 6.0; GraphPad Software Inc., La Jolla, CA, United States).

## Results

### MSC Transplantation Alleviates Liver Failure After Hepatectomy by Promoting Liver Regeneration

**FIGURE 1:**
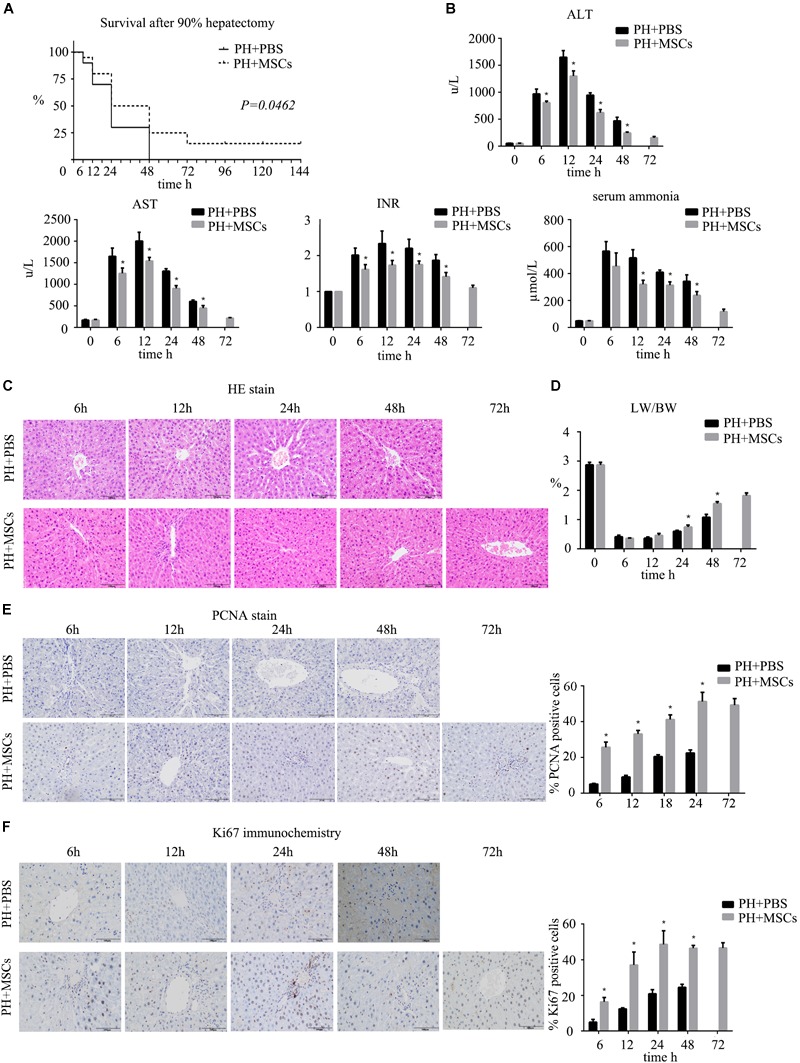
The therapeutic effect of MSCs on PHLF. **(A)** Survival analysis of rats with 90% PH and MSC transplantation. MSC transplantation resulted in a significant decrease in mortality. **(B)** The levels of serum ALT, AST, INR, and ammonia were determined at 6, 12, 24, 48, and 72 h after hepatectomy. MSC transplantation significantly decreased the levels of liver enzymes, INR and ammonia after infusion. **(C)** Liver H&E staining, **(E)** PCNA staining and **(F)** Ki67 staining of each group at 6, 12, 24, 48, and 72 h after hepatectomy (400x). MSC transplantation promoted the hepatocyte proliferation. **(D)** The ratio of liver to body weight. MSC transplantation promoted liver regeneration. ^∗^*P* < 0.05 vs. PH+PBS controls. *t*-test, data are shown as mean ± standard deviation.

Firstly, we established a rat model of PHLF through 90% PH. MSCs were injected into the portal vein after hepatectomy to determine the effect of MSCs on PHLF. Our experiment showed that if the liver failure caused by 90% PH was not treated, all rats would die on the 3rd day. Finally, three rats survived after MSC treatment. However, the survival rate on the 3rd day after 90% PH in the MSC transplantation group was 25% (Figure 1A). As expected, MSCs significantly reduced the levels of ALT, AST, blood ammonia and INR in rats compared with the control group (PBS injection) (Figure 1B). In addition, these parameters peaked after 12 h, with the exception of blood ammonia. Liver histology in rats after hepatectomy was carried out by H&E staining, and MSC transplantation was found to alleviate histologic injury (Figure 1C). Liver regeneration plays a key background role in resuming the liver function. Therefore, we calculated the ratio of liver to body weight and found that MSC transplantation increased such ratio, which was consistent with hepatocellular proliferation in the liver (Figure 1D–F). Moreover, we found that liver regeneration occurred in 6 h, while it was not obvious at the later time points. Hepatocyte proliferation was greater after MSC transplantation compared with the PH+PBS group. Therefore, MSC transplantation had a therapeutic effect on PHLF and promoted liver regeneration.

### MSC Transplantation Improves the Glucose Metabolism After Hepatectomy

**FIGURE 2:**
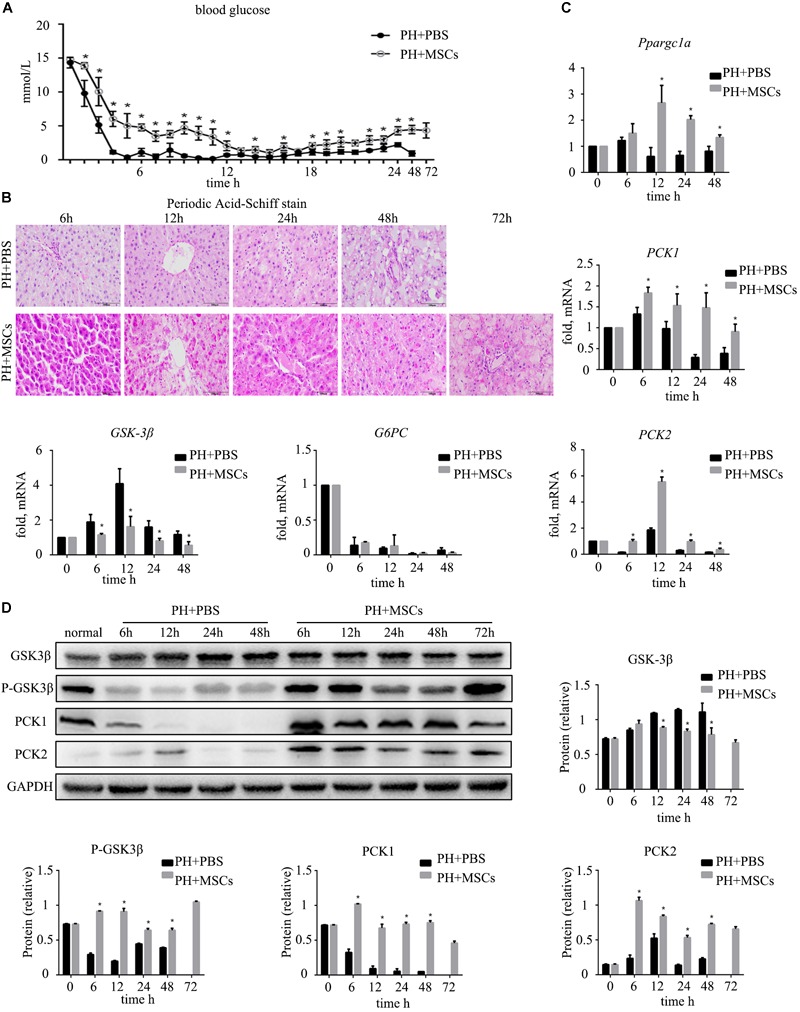
Effect of MSC transplantation on glycometabolism. **(A)** Blood sugar levels were determined within 24 h. MSC transplantation alleviated hypoglycemia. **(B)** PAS staining of liver tissue. Liver glycogen was increased after MSC transplantation. **(C)** Hepatic gene expressions for Ppargc1a, PCK1, PCK2, GSK-3β, and G6PC. **(D)** Western blotting analysis of GSK-3β, p-GSK-3β, PCK1, and PCK2. Liver gluconeogenesis was restored after MSC transplantation. ^∗^*P* < 0.05 vs. PH+PBS controls. *t*-test, data are shown as mean ± standard deviation.

Severe hypoglycemia occurred immediately after 90% PH. Therefore, blood sugar level was monitored for 24 h to observe fluctuations in blood sugar. Interestingly, MSC transplantation enhanced the blood sugar level (Figure 2A), and such enhancement prevented acute hypoglycemia-induced death of rats. It is well known that the liver can regulate glucose metabolism (Postic et al., 2004), and is crucial for maintaining stable blood glucose levels (Petersen et al., 2017). Therefore, we performed PAS staining to determine the glycogen content in the liver. The results were similar to those for blood sugar level, in which glycogen storage was increased after MSC transplantation (Figure 2B). Following MSC transplantation, the status of glucose metabolism disorders was improved. Studies have shown that increased glycogen content in the liver can reduce liver damage after hepatectomy (Tang et al., 2007). It remains unclear how MSC transplantation improves glycogen storage after hepatectomy. Therefore, we examined the expressions of gluconeogenesis-related genes at the mRNA level in the liver. The results showed that the expression of *G6PC* which is related to glycogen decomposition was significantly decreased after hepatectomy, and no significant difference was observed between PH+MSCs group and PH+PBS group. This finding indicated that MSCs increased the glycogen content in the liver mainly by increasing glycogen synthesis rather than reducing glycogen decomposition. In the PH+MSCs group, the expressions of *Ppargc1a, PCK1*, and *PCK2*, which are related to gluconeogenesis, at the mRNA level were up-regulated compared with the PH+PBS group. Our results indicated that GSK-3β, one of the most important kinases which negatively regulate glycogen synthesis, was conspicuously suppressed in the PH+MSCs group (Figure 2C). Considering the obvious difference at the mRNA level, we then examined the expressions of related proteins by Western blotting analysis (Figure 2D). The experimental results indicated that the synthesis of glycogen was dramatically increased (PCK1 and PCK2), while GSK-3β which negatively regulates glycogen synthesis was obviously suppressed. Moreover, we detected the expression of p-GSK-3β^Tyr216^ by Western blotting analysis, while its expression was found to be contrary to that of p-GSK-3β^Ser9^ (Supplementary Figure S1 and Figure 2D).

### MSCs Promote Glycogen Synthesis by AKT/GSK-3β Pathway

**FIGURE 3:**
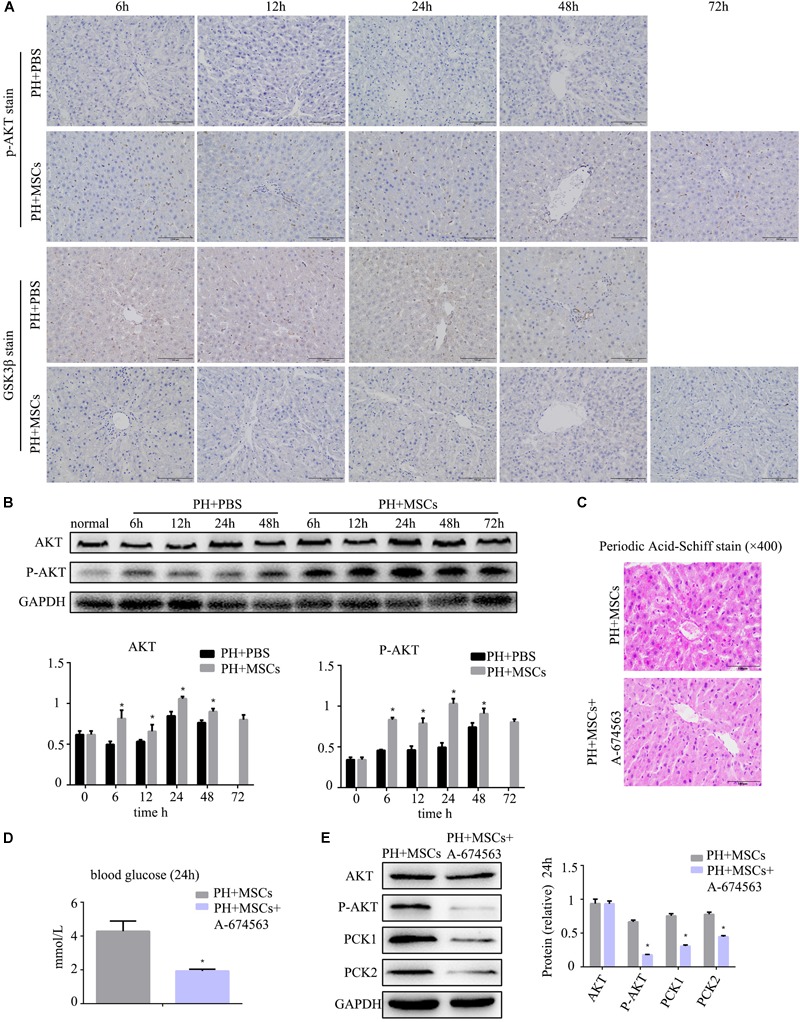
MSC transplantation improves glycogen metabolism by AKT/GSK-3β pathway. **(A)** p-AKT and GSK-3β staining of liver. The expression of p-AKT in the liver was increased, and GSK-3β was decreased. **(B)** Western blotting analysis of AKT and p-AKT. ^∗^*P* < 0.05 vs. PH+PBS controls. The results of Western blotting analysis were consistent with immunohistochemistry staining. **(C)** PAS staining of liver tissue in the PH+MSCs group and PH+MSCs+A-674563 group. Inhibition of AKT counteracted the improvement of glucose metabolism in MSC transplantation. **(D)** Blood sugar levels were determined in 24 h. Inhibition of AKT affected liver glycometabolism. **(E)** Western blotting of AKT, p-AKT, PCK1, and PCK2. ^∗^*P* < 0.05 vs. PH+MSCs controls. *t*-test, data are shown as mean ± standard deviation.

Following 90% PH, the liver requires a lot of energy for liver regeneration, and glycometabolism is impaired, eventually leading to the emergence of hypoglycemia. Severe hypoglycemia stimulates glycogen decomposition, resulting in the release of glucose into the blood. This situation further aggravates liver glucose metabolism disorders. AKT is involved in multiple cellular functions, including growth, proliferation and glucose metabolism. Therefore, we examined the content of p-AKT. MSC transplantation obviously increased the expression of p-AKT (Figure 3A and Supplementary Figure S1C). It was worth considering that the increase of p-AKT expression was accompanied with a decreased GSK-3β level (Figure 3B). Studies have shown that AKT can phosphorylate serine GSK in the 9th position. P-GSK in this state cannot inactivate glycogen synthetase (GS). In the present study, we employed the AKT inhibitor (A-674563) to verify this phenomenon. We found that there was a great difference after MSC treatment at 24 h. Therefore, we selected the time point of 24 h as the observation point. As expected, after the activity of AKT was inhibited, the contents of liver glycogen and blood glucose were significantly decreased (Figure 3C,D). Furthermore, the expressions of key enzymes in liver glycogen synthesis were also down-regulated by AKT inhibitor (A-674563) (Figure 3E). In addition, we found that AKT inhibitor counteracted the effect of MSCs on improving glycometabolism. This finding proved that MSC transplantation improved the glucose metabolism by activating AKT.

### MSCs Promote Cell Proliferation via GSK-3β/β-Catenin Pathway

**FIGURE 4:**
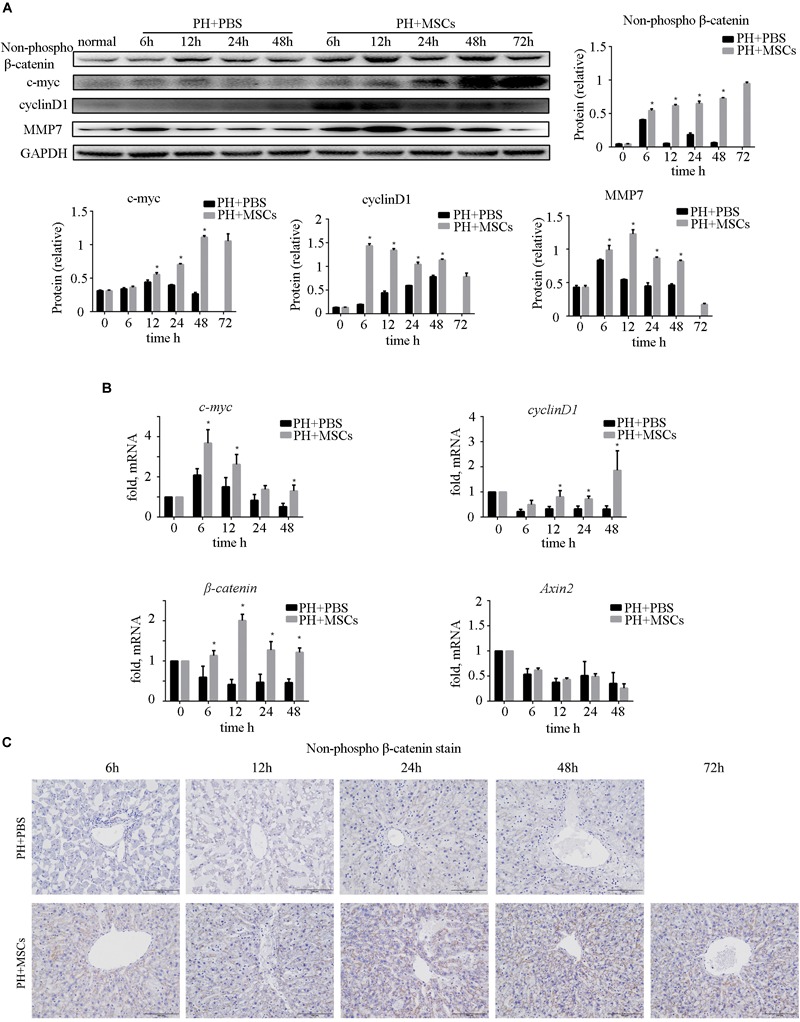
Enhancement of liver regeneration due to MSC transplantation. **(A)** Changes in β-catenin pathway and its downstream protein. β-Catenin pathway was activated by MSC transplantation. **(B)** mRNA changes in β-catenin pathway. **(C)** β-Catenin staining of liver. ^∗^*P* < 0.05 vs. PH+PBS controls. *t*-test, data are shown as mean ± standard deviation.

Many studies have shown that the activation of β-catenin pathway is essential for liver regeneration after hepatectomy (Kawakami et al., 2006; Myung et al., 2007; Bastakoty and Young, 2016). Increased expression of β-catenin induces hepatocyte proliferation and expressions of downstream target genes. We examined the expressions of c-myc and cyclinD1, downstream proteins of β-catenin, and found that their expressions were significantly increased after MSC transplantation (Figure 4A). As an important negative regulatory kinase for glycogen synthesis, GSK-3β can also inhibit β-catenin activity by forming a β-catenin degradation complex. Therefore, we analyzed the expression of non-phospho (active) β-catenin. We found that the content of non-phospho β-catenin was significantly increased after MSC transplantation, which could be correlated with the down-regulation of GSK-3β. In order to assess the biological functions of the nuclear transposition of β-catenin, we detected the expressions of β-catenin and its important downstream genes at the mRNA level by qRT-PCR. We found that the expressions of β-catenin target genes were significantly enhanced (Figure 4B). It further demonstrated that MSC transplantation activated the β-catenin pathway in the liver. Immunohistochemistry was carried out to detect the content of β-catenin in the liver (Figure 4C). Obviously, MSC transplantation increased the content of β-catenin in the liver. We found that after MSC transplantation, β-catenin, which was originally located on the hepatocyte membrane, metastasized to the cytoplasm and nucleus. The activation of β-catenin was different from hepatocellular carcinoma (HCC).

### AKT: A Key Role in MSC Therapy Promotes Cell Proliferation

**FIGURE 5:**
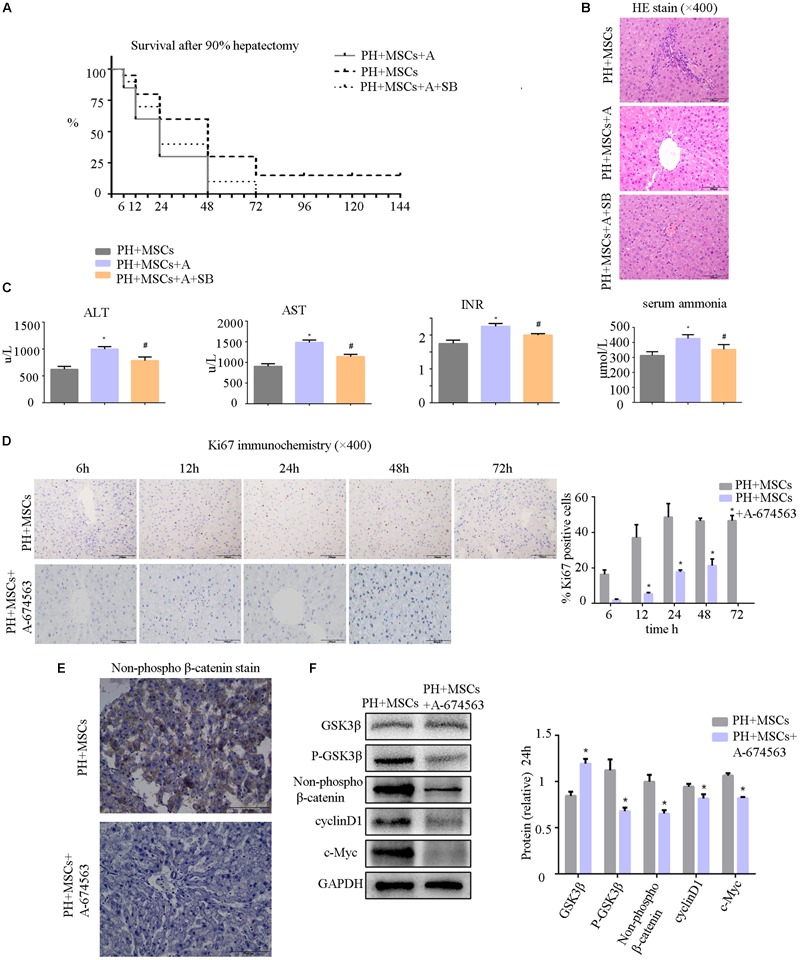
**(A)** Survival analysis of PH+MSCs group, PH+MSCs+A-674563 group and PH+MSCs+SB216763 group. Inhibition of AKT counteracted the therapeutic effect of MSCs. GSK-3β inhibition reduced 90% PH-induced liver injury to a certain extent. **(B)** Liver H&E staining. **(C)** The levels of serum ALT, AST, INR and ammonia were determined at 24 h after hepatectomy. **(D)** Ki67 staining for liver tissue. AKT inhibitor decreased the hepatocyte proliferation. **(E)** β-Catenin staining of liver. **(F)** Western blotting analysis of GSK-3β, p-GSK-3β, β-catenin, cyclinD1, and c-Myc. ^∗^*P* < 0.05 vs. PH+MSCs controls. ^#^*P* < 0.05 vs. PH+MSCs+A-674563 group. *t*-test, data are shown as mean ± standard deviation.

AKT can inhibit the GSK-3β activity by phosphorylating serine in the 9th position of GSK-3β. In the present study, an AKT inhibitor (A-674563) which is upstream of GSK-3β was employed to determine the role of AKT in MSC therapy to PHLF. We found that A-674563 eliminated the therapeutic effect of MSC transplantation. The use of AKT inhibitor increased the mortality of rats undergoing 90% PH and aggravated the liver injury (Figure 5A,B). The levels of ALT, AST, blood ammonia, and INR in rats were significantly increased, suggesting that the degree of liver injury was elevated (Figure 5C). As an important kinase, AKT may play an important role in the regeneration of liver. The inhibitor of AKT not only reversed the therapeutic effect of MSCs, but also aggravated the liver injury. The suppression of GSK-3β alleviated the negative effects of AKT inhibition. Following treatment with A-674563, liver regeneration was obviously inhibited (Figure 5D), and the content of non-phospho β-catenin was decreased in the liver (Figure 5E). Subsequently, we detected the expression of β-catenin downstream protein. Experimental results showed that β-catenin signaling pathway was inhibited as GSK-3β activity was enhanced in the PH+MSCs+A-674563 group (Figure 5F). In addition to improving glucose metabolism, AKT played an important role in the hepatocyte proliferation after MSC transplantation.

Considering the risk of tumor recurrence due to suppression GSK-3β, we assessed the relationship between MSC transplantation and recurrence of HCC. Due to the high mortality rate of 90% PH, we selected 70% PH as the model. We established an HCC tumor model by direct implantation of 10^6^ CBRH-7919 cells into inferior right lobe. We observed the tumorigenesis of rats with 70% PH on the 14th day. Our study supported that MSCs had no effect on the risk of cancer recurrence. There was no significant difference in recurrence rate and size of tumors (Table 2 and Supplementary Figure S1B). Although MSC transplantation inhibited the expression of GSK-3β, it didn’t increase the risk of HCC recurrence.

**Table 2:** Tumor growth in the different rat HCC model groups.

	PH+PBS	PH+MSCs
Tumor forming	4/6	4/6
The number of tumor nodules, mean ± SEM	1.25 ± 0.25	1.25 ± 0.25
The largest diameter of tumors, mean ± SEM (mm)	97.9 ± 6.43	99.7 ± 4.17

*SEM, standard error of mean.*

## Discussion

Liver is the largest metabolic organ in the human body (Kampf et al., 2014). Liver disease can affect liver metabolism, and changes in liver metabolism affect the development of liver diseases (Kietzmann, 2017). The liver plays a significant role in glucose metabolism, which is vital in maintaining the blood sugar balance. Previous studies have found that MSCs have immunomodulatory functions and can reduce hyperinflammatory lesions (Natsumeda et al., 2017; Willis et al., 2018). In the present study, we found that MSCs had not only metabolic regulatory functions but also capabilities of liver regeneration. Hepatectomy with massive hepatic volume will lead to postoperative hepatic function failure. Our experiment showed that if liver failure caused by 90% PH was not treated, all rats would die on the 3rd day. The survival rate of rats after 70% PH was almost 100% even in obese rats. Compared with 70% PH, 90% PH is a more accurate representation of acute liver failure after hepatectomy. Therefore, we selected 90% PH in rats as an experimental model to induce acute liver failure (Zhang et al., 2015). Hypoglycemia often occurs immediately after 90% PH, which may trigger liver regeneration after hepatectomy (Gazit et al., 2012; Miyaoka and Miyajima, 2013; Kachaylo et al., 2017). Although hypoglycemia can stimulate liver regeneration, sustained rapid hypoglycemia without treatment can cause death in rats (Reno et al., 2018). We confirmed that marked hypoglycemia occurred after 90% PH. Liver glycogen was significantly reduced after 90% PH since hypoglycemia can result in breakdown of liver glycogen to maintain blood glucose. However, an opposite finding was observed after MSC transplantation. The content of liver glycogen was significantly increased after MSC transplantation in the liver failure model with 90% PH, and hypoglycemia symptoms were also attenuated. Surviving rats showed corrected severe hypoglycemia. Our previous study has shown that MSCs are recruited to injured liver after transplantation (Ma et al., 2014). Recently studies support the role of paracrine mechanism in MSC transplantation therapy (Chen et al., 2008; Zagoura et al., 2012). However, it still remains unclear which cytokines are responsible for liver repair. We will reveal specific molecular mechanisms in the future study. Moreover, it is unclear how MSCs improve glucose metabolism in the liver. Therefore, we examined the expressions of genes related to glycoprotein synthesis at the protein and mRNA levels, and found that the function of GSK-3β was suppressed after MSC transplantation.

As a widely expressed serine/threonine kinase, GSK-3 is first identified as a key enzyme involved in glycogen metabolism (Embi et al., 1980). GSK-3 prevents glycogen synthesis by phosphorylating GS (Halse et al., 1999; Doble and Woodgett, 2003). GSK-3 consists of 433 amino-acid residues and contains two subtypes of GSK-3α and GSK-3β in mammals (Doble and Woodgett, 2007). These two subtypes have similar conformation and zymolyte, but their functions are different. GSK-3β is widely found in all types of cells (Woodgett, 1991). As a downstream regulator, GSK-3β affects glycogen metabolism, participates in a variety of signal transduction pathways and plays an important role in embryo development, cell differentiation and tumor formation (Doble and Woodgett, 2003; Jope and Johnson, 2004). We found that MSC transplantation significantly inhibited the activity of GSK-3β by increasing AKT activity to phosphorylate serine in the 9th position of GSK-3β. Subsequently, suppressed GSK-3β prevented the formation of β-catenin degradation complex, which is composed of β-catenin, APC, Axin, GSK-3β and CK1α (Larue and Delmas, 2006), and free β-catenin can accumulate in the cytoplasm (Wu et al., 2009). When β-catenin is accumulated to a certain level in the cytoplasm, it is transferred to the nucleus to play a role in promoting cell proliferation and growth. β-Catenin has been shown to be abnormally expressed in breast cancer, pancreatic cancer, colorectal cancer, and other malignant tumor cells. β-Catenin plays an important role in the regeneration of damaged tissues (Kawakami et al., 2006). Many genes regulating cell proliferation, differentiation and tumorigenesis are regulated by the β-catenin pathway, such as c-myc and cyclinD1. These two genes can accelerate G1/S phase in the cell cycle and promote cell growth and proliferation. The β-catenin pathway is crucial for liver regeneration in liver diseases. Transposition of β-catenin into the nucleus increases the expressions of c-myc and cyclinD1, which are associated with cell proliferation (Larue and Delmas, 2006).

To confirm the important role of AKT/GSK-3β in the treatment of PHLF by MSCs, we used an AKT inhibitor to activate GSK-3β by suppressing the phosphorylation of GSK-3β (Chen et al., 2017). We found that when GSK-3β was activated, the therapeutic effect of MSCs was eliminated. AKT plays an important role in regulating cell function, such as metabolism, growth, proliferation, survival, transcription, and protein synthesis. Our study found that the phosphorylation of AKT was increased after MSC treatment, leading to affected function of GSK3β and improved glucose metabolism. The proliferation of hepatocytes is very important for the liver regeneration. Our finding that MSCs promoted liver regeneration might be of clinical value. The most common causes of death after liver surgery are PHLF and small liver syndrome. Increasing the recovery ability of the liver by promoting the hepatocyte proliferation could reduce the occurrence of these complications.

Collectively, we studied the effect of MSC transplantation on liver failure after hepatectomy. We found that MSCs had a positive effect on liver failure after hepatectomy. These results emphasized that AKT/GSK-3β pathway played an important role in liver regeneration after hepatectomy. MSCs increased liver glycogen synthesis by inhibiting GSK-3β. More importantly, MSC transplantation increased cell proliferation via the GSK-3β/β-catenin pathway. However, these preliminary findings were only based on animal experiments. Therefore, preclinical studies are necessary to verify the current findings.

## Ethics Statement

This study was carried out in strict accordance with the recommendations in the Guide for the Care and Use of Laboratory Animals of the National Institutes of Health. The protocol was approved by the Committee on the Ethics of Animal Experiments of the Nanjing Drum Tower Hospital (Approval No. SYXK2014-0052). All surgeries were performed under chloral hydrate anesthesia, and all efforts were made to minimize animal suffering.

## Author Contributions

H-rD and J-lW conceived and designed the study, collected and assembled the data, performed the data analysis and interpretation, and wrote the manuscript. Z-tT and YW conceived and designed the study, collected data, and wrote the manuscript. H-zR and X-lS conceived and designed the study, provided the financial support and study material, performed the data analysis and interpretation, wrote and gave the final approval of the manuscript. All authors read and approved the manuscript.

## Conflict of Interest Statement

The authors declare that the research was conducted in the absence of any commercial or financial relationships that could be construed as a potential conflict of interest.

## Supplementary Material

FIGURE S1 **(A)** P-GSK-3β^Tyr216^ decreased after MSCs transplantation, which was opposed to P-GSK-3β^ser9^. **(B)** The transplantation of MSCs had no significant effect on the growth of HCC. **(C)** In order to present the results of histochemistry stain of p-AKT and GSK-3β, we calculated the Mean of IOD through image Pro Plus software (Media Cybernetics, Bethesda, MD, United States). **(D)** Western Blot of Non-phospho β-catenin. **(E)** Ki67 stain for liver tissue.

## Abbreviations

ALT: alanine transaminase
APC: adenomatous polyposis coli
AST: aspartate aminotransferase
CK1α: casein kinase 1α
GSK-3β: glycogen synthase kinase 3β
GSK-3: glycogen synthase kinase 3
IL-6: interleukin-6
INR: international normalized ratio
KI67: antigen KI67
MSCs: mesenchymal stem cells
PAS: Periodic Acid-Schiff
PBS: phosphate buffered saline
PCK1: phosphoenolpyruvate carboxykinase 1
PCK2: phosphoenolpyruvate carboxykinase 2
PH: partial hepatectomy
PHLF: acute liver failure after partial hepatectomy
SD rats: Sprague-Dawley rats
TNF-α: tumor necrosis factor-α
