# Supplementary figures and images for: Mesenchymal Stem Cells Improve Glycometabolism and Liver Regeneration in the Treatment of Post-hepatectomy Liver Failure
Source: Front Physiol. 2019 Apr 10;10:412. doi: 10.3389/fphys.2019.00412 (PMC6468048; doi:10.3389/fphys.2019.00412)

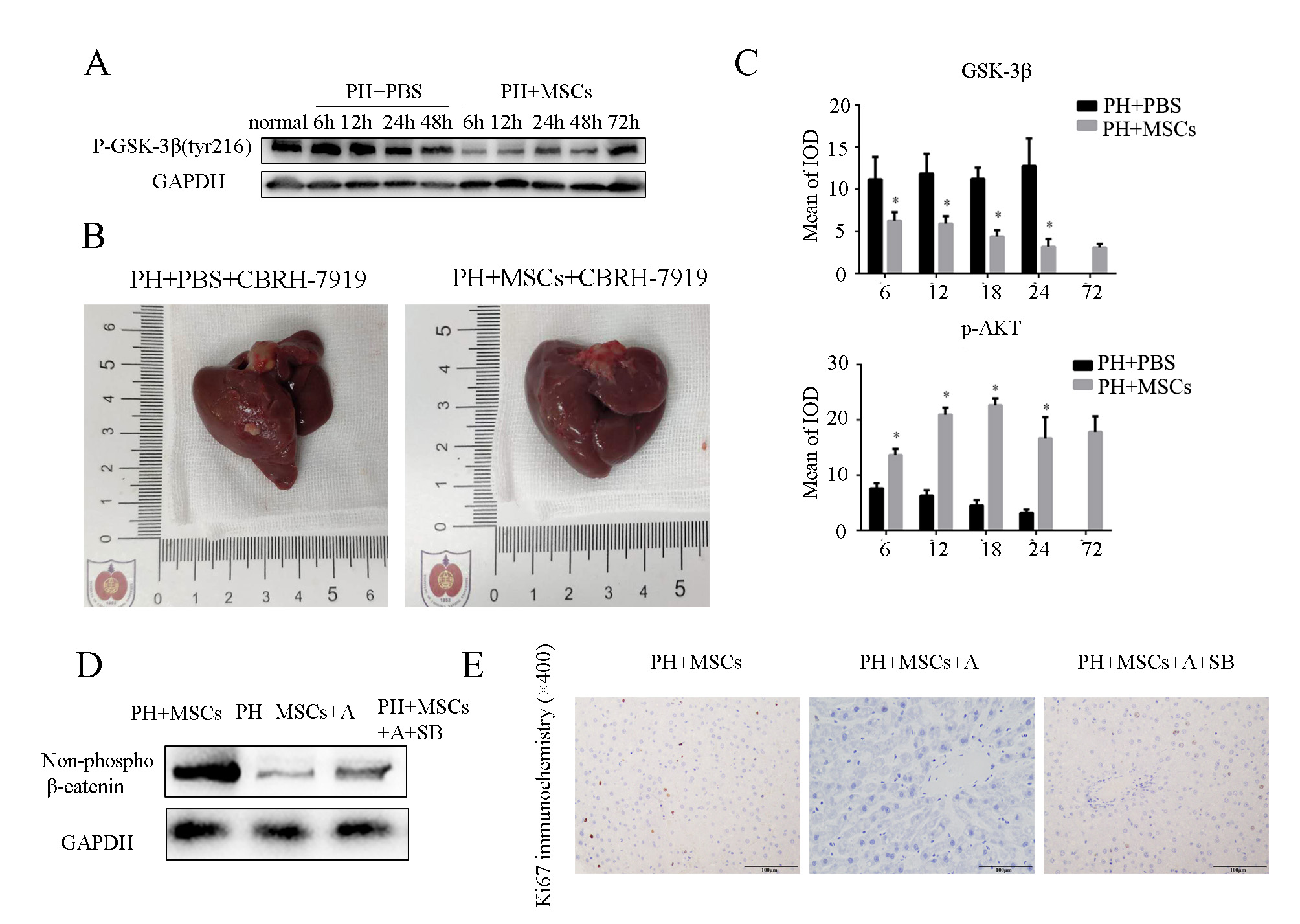

Supplement: FIGURE S1 — (A) P-GSK-3βTyr216 decreased after MSCs transplantation, which was opposed to P-GSK-3βser9. (B) The transplantation of MSCs had no significant effect on the growth of HCC. (C) In order to present the results of histochemistry stain of p-AKT and GSK-3β, we calculated the Mean of IOD through image Pro Plus software (Media Cybernetics, Bethesda, MD, United States). (D) Western Blot of Non-phospho β-catenin. (E) Ki67 stain for liver tissue. [file Image_1.jpg]
